# Editorial: The Belt and Road of Animal Diseases

**DOI:** 10.3389/fvets.2021.795556

**Published:** 2021-11-08

**Authors:** Shao-Lun Zhai

**Affiliations:** ^1^Institute of Animal Health, Guangdong Academy of Agricultural Sciences, Guangzhou, China; ^2^Scientific Observation and Experiment Station of Veterinary Drugs and Diagnostic Techniques of Guangdong, Ministry of Agriculture of Rural Affairs, Guangzhou, China; ^3^Key Laboratory of Animal Disease Prevention of Guangdong, Guangzhou, China; ^4^Guangdong Laboratory for Lingnan Modern Agriculture, Maoming Branch, Maoming, China

**Keywords:** the belt and road, transboundary animal infectious diseases, exotic animal, spread, transmission

## Introduction

The Belt and Road Initiative (BRI) is a global development strategy adopted by the Chinese government in 2013. This investment and infrastructure development strategy drives the world's economic development. However, this initiative has resulted in an increase in the transnational spread of animal diseases, which caused a significant economic loss to the global animal industry. For example, in 2013, Peste des petits ruminant virus entered China (1); in 2015, Senecavirus A outbreak occurred in China (2); since 2018, African swine fever virus swept into east Asia and Southeast Asia (3); in 2019; lumpy skin disease virus emerged in China (4); in 2013, Porcine Epidemic Diarrhea was found in the United States (5). Strategies to avoid or reduce transnational spread of animal infectious diseases is an important issue, which can be addressed by a multidisciplinary approach including rapid virus detection, metagenomic sequencing, and epidemiology. Other measures such as scientific popularization of disease knowledge, combating smuggling activities, border trade control, and wildlife management should facilitate the control and prevention of these rapidly evolving infectious diseases of animals in a timely and effective manner.

The aim of this Research Topic is to offer an opportunity to collect the newest research and development in the field of “The Belt and Road” of Animal Diseases. Early warning, virus detection, genetic evolution, disseminating disease knowledge, combating smuggling activities, border trade control (among others) are topics that we aim to explore.

## Organization of the Research Topic

In this Research Topic, we received 16 manuscripts, 11 (1 review, 9 original research, 1 brief research report) were accepted for publication. Among them, 9 papers involved virus research, and two papers involved bacterial research. In 9 viral topic-related papers, 5 papers involved nidovirus research; 2 papers involved circovirus research; one paper involved herpesvirus research; one paper focused on picornavirus research. The first nidovirus paper reported the genetic diversity (Genotype 1, 2, 3) of ORF3 gene of porcine epidemic diarrhea virus (PEDV) in Guangxi, China, more specifically, the continuous deletions from 172 to 554 bp of ORF3 gene occurred in some PEDV strains (Lu et al.). The second nidovirus research paper described a re-outbreak of PEDV with 97.8% similarity with the vaccine strain CV777 in one vaccinated farm, indicating that current commercial PEDV vaccine could not fully protect pigs against the epidemic strains (Gao et al.). The two papers all emphasized the research and development of an effective and broad-spectrum PEDV vaccine. The third nidovirus study showed that porcine enteric alphacoronavirus (PEAV) could inhibit IFN-α, IFN-β, OAS, Mx1, and PKR mRNA expression in infected peyer's patches *in vivo* (Xu, Gong, et al.). The forth nidovirus paper, a review article, mainly summarized the apoptosis roles in swine response to viral infection and pathogenesis of four swine coronaviruses including PEDV, PEAV, transmissible gastroenteritis virus (TGEV), and porcine deltacoronavirus (PDCoV) (Xu, Zhang, et al.). The two papers revealed the pathogenesis of viral infections and laid the foundation to develop an appropriate strategy for the prevention and control of swine diarrhea diseases. The fifth nidovirus paper described a monoclonal antibody against porcine CD163 SRCR5 domain, which partially could block the infection of porcine reproductive and respiratory syndrome virus (PRRSV) (Zhang et al.). It may provide a foundation of antiviral therapy for PRRSV. In two circovirus studies, the authors established two different methods to detect porcine circovirus 3 (PCV3). One was colorimetric isothermal multiple-self-matching-initiated amplification assay, which might be a good practical choice for epidemiological investigation and point-of-care testing of PCV3 (Gou et al.). Another was single multiple cross displacement amplification assay, which could be widely applied in PCV3 detection in laboratories or in rural areas (Bian et al.). During eighth virus theme-related study, Xu, Gao, Wu, et al. identified that UL46 gene of pseudorabies virus (PRV) could encode the nucleocytoplasmic shuttling protein. In the final paper, Zhou et al. first reported seneca valley virus (SVV) in buffalos in China, and sequence analysis showed the SVV strain was close to one wild boar-origin strain, which revealed potential cross-species transmission of SVV.

In two bacterial studies, Liu et al. collected genotype data of Brucella strains from 11 countries along the Silk Road, the combination of whole-genome sequencing and single-nucleotide polymorphism (WGS-SNP) phylogenetic analysis showed that some genotypes spread within the national borders and other genotypes continuously expanded and spread in countries along with Silk Road. The authors strengthen that there is an urgent need for the control (especially entry-exit quarantine of animal brucellosis) of transfer and trade of infected sheep/goats in countries along the Silk Road. Li et al. reported the host cytokine response differences in piglets infected with toxigenic and non-toxigenic staphylococcus hyicus (S. hyicus) It was helpful to understand the pathogenic mechanisms of S. hyicus.

## Conclusion

Since the beginning of 2020, The Research Topic began to receive the manuscript submission, and invited more than 60 research team from the world to submit the manuscript. But we finally received 16 manuscripts that might be caused on on-going COVID-19 pandemic, which clearly affects our research and work progress. Although the Topic provides overviews of some pathogen cross-region threats and discusses current and novel strategies for the detection and control, the manuscripts associated with transboundary infectious diseases and foreign diseases (especially Peste des petits ruminant, African swine fever and lumpy skin disease) were lacking. In the following work, we hope that more scientists pay more attention to these diseases in order to reduce or prevent the spread of transboundary infectious diseases and foreign diseases in animals.

## Author Contributions

S-LZ wrote the editorial and approved it for publication.

## Funding

This work was supported by the two grants (Nos. 2019B020211005 and 2019B020217002) from the Guangdong Science and Technology Department, the Jinying's Star Talent Program (Grant No. R2018PY-JX003) and Disciplinary Team Construction Program (Grant No. 202122TD) from Guangdong Academy of Agricultural Sciences, the grant (No. 201906040005) from Guangzhou Science and Technology Bureau, and the two grants (Nos. 2021KJ114 and 2021KJ119) from the Department of Agriculture and Rural Affairs of Guangdong Province.

## Conflict of Interest

The author declares that the research was conducted in the absence of any commercial or financial relationships that could be construed as a potential conflict of interest.

## Publisher's Note

All claims expressed in this article are solely those of the authors and do not necessarily represent those of their affiliated organizations, or those of the publisher, the editors and the reviewers. Any product that may be evaluated in this article, or claim that may be made by its manufacturer, is not guaranteed or endorsed by the publisher.

## References

[1] Bao J Wang Q Zhang Y Liu C Li L Wang Z. Complete genome sequence of a novel variant strain of peste des petits ruminants virus, China/XJYL/2013. Genome Announc. (2014) 2:E00762–14. 10.1128/GenomeA.00762-14 25301639 PMC4192371

[2] Wu Q Zhao X Bai Y Sun B Xie Q Ma J. The first identification and complete genome of senecavirus a affecting pig with idiopathic vesicular disease in China. Transbound Emerg Dis. (2017) 64:1633–40. 10.1111/Tbed.12557 27539949

[3] Mighell E Ward MP. African swine fever spread across Asia, 2018-2019. Transbound Emerg Dis. (2021) 68:2722–32. 10.1111/tbed.14039 33599077

[4] Lv DH Zhai SL Wei WK Zhai Q Wen XH Chen QL. Threat of lumpy skin disease to the Chinese cattle industry. Vet Rec. (2021) 188:315–6. 10.1002/vetr.434 33891759

[5] Williamson S Strugnell B Thomson J Webster G McOrist S Clarke H. Emergence of severe porcine epidemic diarrhoea in pigs in the USA. Vet Rec. (2013) 173:146–8. 10.1136/vr.f4947 23934297

